# Supplementary material for: Distillation of photon entanglement using a plasmonic metamaterial
Source: Sci Rep. 2015 Dec 16;5:18313. doi: 10.1038/srep18313 (PMC4680945; doi:10.1038/srep18313)
Supplement: Supplementary Information [file srep18313-s1.pdf]

Supplementary Information: Distillation of photon entanglement using a plasmonic metamaterial

Motoki Asano[1], Muriel Bechu[2,3], Mark Tame[4,5], Şahin Kaya Özdemir [6*], Rikizo Ikuta[1], Durdu Ö. Güney[7], Takashi Yamamoto[1], Lan Yang[6], Martin Wegener[2,3*] and Nobuyuki Imoto[1*]

[1]Department of Material Engineering Science, Graduate School of Engineering Science, Osaka University, Toyonaka, Osaka 560-8531, Japan.

[2]Institute of Applied Physics, Karlsruhe Institute of Technology (KIT), 76128 Karlsruhe, Germany.

[3]Institute of Nanotechnology, Karlsruhe Institute of Technology (KIT), 76128 Karlsruhe, Germany.

[4]School of Chemistry and Physics, University of KwaZulu-Natal, Durban 4001, South Africa.

[5]National Institute for Theoretical Physics, University of KwaZulu-Natal, Durban 4001, South Africa.

[6]Department of Electrical and Systems Engineering, Washington University, St. Louis, MO 63130, USA.

[7]Department of Electrical and Computer Engineering, Michigan Technological University, Houghton, MI 49931, USA.

**Correspondence to: imoto@mp.es.osaka-u.ac.jp, ozdemir@wustl.edu, martin.wegener@kit.edu*

1. Metamaterial fabrication

The metamaterials were fabricated by electron-beam lithography followed by a lift-off procedure. Precisely, 5 mm × 5 mm suprasil substrates were prepared for electron-beam lithography by depositing a 5 nm thin layer of indium tin oxide (ITO) by electron-beam evaporation. Next, an approximately 200 nm thick film of polymethylmetacrylate photoresist (MicroChem) was spin-coated on top of the ITO. This positive tone photoresist was then patterned by electron-beam writing (Raith e-line). We wrote 160 different arrays of straight nano-antennas. The nano-antenna length and thickness was varied between the arrays to shift

the spectral position of the resonance. Each array has a footprint of 100 μm × 100 μm and contains antennas with a horizontal spacing of 200 nm and nominal lengths between 95 nm and 110 nm. After exposure, the samples were developed in a 1:3 solution of methyl isobuthyl ketone (MIBK) and isopropanol. This process dissolves the long-chained molecules of the photoresist that have been broken up during the exposure process and thus creates a mask. Onto this mask, 30 nm of gold were deposited by high-vacuum electron-beam evaporation. To lift off the PMMA mask and the excess gold, the samples were exposed to a bath of Allresist remover AR 300-70 at 50° C until the lift-off was completed.

2. Quantum process tomography

The general form of a quantum channel corresponding to a completely positive map on the state ρ is given by $\rho \rightarrow \sum_{ij} \chi_{ij} E_i \rho E_j^{\dagger}$, where $\sum_i E_i^{\dagger} E_i \leq I$ (with equality for a trace-preserving map). For a single qubit, the Pauli operators, $E_i = I, X, Y$ and Z, provide a complete basis for the Hilbert space. For the ideal model of a partial polarizer given in the main text we have the channel $\rho \rightarrow K_0 \rho K_0^{\dagger}$, where the Kraus operator $K_0 = |H\rangle\langle H| + \sqrt{T_V}|V\rangle\langle V|$ corresponds to a non-trace preserving channel. The equivalent form of this channel in the Pauli basis is given by the general map $\varepsilon: \rho \rightarrow \sum_{ij} \chi_{ij} E_i \rho E_j^{\dagger}$, where the elements of the χ matrix are

$$\chi_{id} = \begin{pmatrix} (1+2\sqrt{T_V}+T_V)/4 & 0 & 0 & (1-T_V)/4 \\ 0 & 0 & 0 & 0 \\ 0 & 0 & 0 & 0 \\ (1-T_V)/4 & 0 & 0 & (1-2\sqrt{T_V}+T_V)/4 \end{pmatrix} \quad \text{(S1)}$$

which gives $\mathrm{Tr}(\chi) = (1+T_V)/2$. This channel is trace preserving (and unitary) only for $T_V = 1$. In order to obtain the elements of an experimental χ matrix for a given single-qubit channel ε, one can probe it with the four states $|H\rangle$, $|V\rangle$, $|D\rangle$ and $|R\rangle$, which allow the reconstruction of the action of ε on the different elements of an arbitrary input state: $\varepsilon(|H\rangle\langle H|)$, $\varepsilon(|V\rangle\langle V|)$, $\varepsilon(|H\rangle\langle V|) = \varepsilon(|D\rangle\langle D|) + i\varepsilon(|R\rangle\langle R|) - \frac{1}{2}(1+i)[\varepsilon(|H\rangle\langle H|) + \varepsilon(|V\rangle\langle V|)]$ and $\varepsilon(|V\rangle\langle H|) = \varepsilon(|D\rangle\langle D|) - i\varepsilon(|R\rangle\langle R|) - \frac{1}{2}(1-i)[\varepsilon(|H\rangle\langle H|) + \varepsilon(|V\rangle\langle V|)]$. From this it is straightforward to extract out the χ matrix elements [1]. To obtain the different probe state outputs $\varepsilon(|i\rangle\langle i|)$, we prepare each of the probe states and send them into the metamaterial. The

output states are then obtained from quantum state tomography. Note, however, that the channel is expected to be non-trace preserving. Thus we must weight the different output states by their relative probability of being produced, given a probe state was input to the channel. For instance, the probe state $|V\rangle$ is only expected to be transmitted through the metamaterial with probability T_V in the ideal case, thus the output state $\varepsilon(|V\rangle\langle V|) = |V\rangle\langle V|$ would be produced with probability T_V and any channel reconstruction would need to weight $\varepsilon(|V\rangle\langle V|)$ by the factor T_V. More generally, for a fixed time period we count the number of output states transmitted by a given input probe state when there is no metamaterial present (glass substrate only). This is obtained by measuring the total number of counts for measurements in the $|H\rangle/|V\rangle$ basis, providing a reference value, N_i^r, for each probe state i when there is no metamaterial (corresponding to the identity operation). In the presence of the metamaterial we again count the number of output states transmitted by a given probe state using the $|H\rangle/|V\rangle$ basis, which provides the value N_i. The relative probability of an output state being produced by the metamaterial given a probe state was input is then given by $p_i = N_i/N_i^r$. This weighting of the probe state outputs $\varepsilon(|i\rangle\langle i|)$ leads to a non-trace preserving χ matrix. In Fig. S1, we show the reconstructed χ matrices for the seven different metamaterials studied in our experiment. The process fidelity $F_P = \mathrm{Tr}\left(\sqrt{\sqrt{\chi}\chi_{id}\sqrt{\chi}}\right)^2 / \mathrm{Tr}(\chi)\mathrm{Tr}(\chi_{id})$ of each χ matrix with respect to the ideal partial polarizer χ_{id} is maximized over the variable T_V, leading to the ideal χ_{id} matrices shown to the right of the corresponding experimentally reconstructed ones. The process fidelities are given in the caption along with the maximized T_V values.

3. Density matrices of distilled states for pure input states

In Figure S2 we show the density matrices of the distilled states from each of the seven metamaterials.

4. Density matrices of distilled states for non-maximally entangled partially mixed input states

To prepare non-maximally entangled partially mixed states in our experiment, we implement a phase damping channel by using a quartz plate sandwiched between two HWPs inserted into

the path of one of the photons. The quartz plate induces phase damping in the polarization basis by introducing a delay between photons with horizontal polarization and those with vertical polarization. This delay is comparable to the coherence time of the two terms in the non-maximally entangled input state but much shorter than the coincidence window and therefore produces an effective phase damping effect. The HWPs enable the amount of phase damping to be controlled by rotating the polarization basis in which the phase damping occurs. The Kraus operators corresponding to this optical configuration are given by

$$E_1(\theta,\lambda) = U_{HWP}^{-1}(\theta)\begin{pmatrix}1 & 0\\ 0 & \sqrt{1-\lambda}\end{pmatrix}U_{HWP}(\theta)\otimes I, \tag{S2}$$

$$E_2(\theta,\lambda) = U_{HWP}^{-1}(\theta)\begin{pmatrix}0 & 0\\ 0 & \sqrt{\lambda}\end{pmatrix}U_{HWP}(\theta)\otimes I, \tag{S3}$$

where $U_{HWP}(\theta) = \begin{pmatrix}\cos\theta & \sin\theta\\ \sin\theta & -\cos\theta\end{pmatrix}$ and I is the identity operation. Here, each matrix is written in the $|H\rangle/|V\rangle$ basis, λ represents the degree of phase damping corresponding to the difference of group velocity between each polarization (due to the quartz plate). Using the Kraus representation, the partially mixed state produced by acting on the non-maximally entangled state $|\Phi_\varepsilon\rangle$ is expressed by [2]

$$\rho_{in}(\theta,\lambda,\varepsilon) = \frac{E_1|\Phi_\varepsilon\rangle\langle\Phi_\varepsilon|E_1^\dagger + E_2|\Phi_\varepsilon\rangle\langle\Phi_\varepsilon|E_2^\dagger}{Tr[E_1|\Phi_\varepsilon\rangle\langle\Phi_\varepsilon|E_1^\dagger + E_2|\Phi_\varepsilon\rangle\langle\Phi_\varepsilon|E_2^\dagger]} = \frac{1}{1+\varepsilon^2}\begin{pmatrix}a_1 & a_2 & a_4 & a_7\\ a_2 & a_3 & a_5 & a_8\\ a_4 & a_5 & a_6 & a_9\\ a_7 & a_8 & a_9 & a_{10}\end{pmatrix} \tag{S4}$$

where $|\Phi_\varepsilon\rangle$ corresponds to the pure state defined in Eq. (1), and each element is exactly calculated as the following:

$$a_1 = \varepsilon^2\left(1 - 2\cos^2\theta\sin^2\theta\left(1-\sqrt{1-\lambda}\right)\right) \tag{S5}$$

$$a_2 = \frac{\varepsilon}{4}\sin 4\theta\left(1-\sqrt{1-\lambda}\right)$$

$$a_3 = 2\cos^2\theta\sin^2\theta\left(1-\sqrt{1-\lambda}\right)$$

$$a_4 = \frac{\varepsilon^2}{4}\sin 4\theta\left(1-\sqrt{1-\lambda}\right)$$

$$a_5 = 2\varepsilon\cos^2\theta\sin^2\theta\left(1-\sqrt{1-\lambda}\right)$$

$$a_6 = \frac{\varepsilon^2}{2}\sin^2 2\theta\left(1-\sqrt{1-\lambda}\right)$$

$$a_7 = \frac{\varepsilon}{4}\left(1+3\sqrt{1-\lambda}-\left(1-\sqrt{1-\lambda}\right)\cos 4\theta\right)$$

$$a_8 = -\frac{1}{4}\sin 4\theta\left(1-\sqrt{1-\lambda}\right)$$

$$a_9 = -\frac{\varepsilon}{4}\sin 4\theta\left(1-\sqrt{1-\lambda}\right)$$

$$a_{10} = 1-2\cos^2\theta\sin^2\theta\left(1-\sqrt{1-\lambda}\right).$$

We can obtain a simple approximate form of the density matrix for the non-maximally entangled partially mixed state by omitting terms that are higher than the second order of λ and θ, and cross terms to first order,

$$\rho_{in}(\lambda,\varepsilon) \approx \frac{1}{1+\varepsilon^2}\begin{bmatrix} \varepsilon^2|HH\rangle\langle HH| + |VV\rangle\langle VV| \\ +\varepsilon\left(1-\frac{\lambda}{2}\right)(|HH\rangle\langle VV| + |VV\rangle\langle HH|)\end{bmatrix} \tag{S6}$$

The final state is then approximately given by

$$\rho_{out}(\lambda,\varepsilon) = \frac{K_0\rho_{in}(\lambda,\varepsilon)K_0^\dagger}{\mathrm{Tr}\left[K_0\rho_{in}(\lambda,\varepsilon)K_0^\dagger\right]} \tag{S7}$$

$$\approx \frac{1}{T_V+T_H\varepsilon^2}\left[\varepsilon^2 T_H|HH\rangle\langle HH| + T_V|VV\rangle\langle VV| + \varepsilon\sqrt{T_H T_V}\left(1-\frac{\lambda}{2}\right)(|HH\rangle\langle VV + |VV\rangle\langle HH|)\right]$$

where K_0 represents the Kraus operator of the metamaterial. We obtained the experimental parameters λ_{exp} and ε_{exp} summarized in Table 1 using the following relations given by the approximate form of the density matrices

$$\varepsilon_{exp} = \sqrt{\frac{\mathrm{Tr}[\rho_{exp}|HH\rangle\langle HH|]}{\mathrm{Tr}[\rho_{exp}|VV\rangle\langle VV|]}}, \tag{S8}$$

$$\lambda_{exp} = 2\left(1-\frac{\mathrm{Tr}[\rho_{exp}|HH\rangle\langle VV|]}{\mathrm{Tr}[\rho_{exp}|HH\rangle\langle HH|]}\varepsilon_{exp}\right), \tag{S9}$$

where ρ_{exp} represents the experimentally reconstructed density matrix in the distillation of partially mixed states. A similar derivation to the above can be performed for the non-maximally entangled partially mixed initial state for $|\Psi_\varepsilon\rangle$. Figure S3 shows all the density matrices of the initial partially mixed states and final distilled states that are summarized in

Table 1 in the main text. In the case where θ is small, the experimental density matrices have four dominant components as expected from the theoretical approximation. On the other hand, there are several additional components in the case of large θ.

1. I. L. Chuang and M. A. Nielsen, “Prescription for experimental determination of the dynamics of a quantum black box,” J. Mod. Opt. **44**, 2455 (1997).

2. M. A. Nielsen and I. L. Chuang, Quantum Computation and Quantum Information (Cambridge University Press, Cambridge, 2010).

Figure S1. Characterization of metamaterials by quantum process tomography. Experimentally obtained process matrices (χ matrices) for the different metamaterials used in the experiment for entanglement distillation. The process matrices are given in the basis defined by the single-qubit Pauli operators, E_i = I, X, Y and Z, where a single qubit is modified as $\rho \to \sum_{ij} \chi_{ij} E_i \rho E_j^{\dagger}$. (a) Metamaterial sample 1 (left) and an ideal partial polarizer with $T_V = 0.11 \pm 0.01$ (right). The process fidelity of the sample to the ideal case is 0.93±0.01 (Tr(χ)=0.53±0.01). (b) Metamaterial sample 2 (left) and an ideal partial polarizer with $T_V = 0.13 \pm 0.01$ (right). The process fidelity of the sample to the ideal case is 0.92±0.02 (Tr(χ)=0.55±0.01). (c) Metamaterial sample 3 (left) and an ideal partial polarizer with $T_V = 0.16 \pm 0.01$ (right). The process fidelity of the sample to the ideal case is 0.95±0.01 (Tr(χ)=0.54±0.01). (d) Metamaterial sample 4 (left) and an ideal partial polarizer with $T_V = 0.21 \pm 0.01$ (right). The process fidelity of the sample to the ideal case is 0.94±0.01 (Tr(χ)=0.56±0.01). (e) Metamaterial sample 5 (left) and an ideal partial polarizer with $T_V = 0.27 \pm 0.01$ (right). The process fidelity of the sample to the ideal case is 0.92±0.02 (Tr(χ)=0.60±0.01). (f) Metamaterial sample 6 (left) and an ideal partial polarizer with $T_V = 0.41 \pm 0.01$ (right). The process fidelity of the sample to the ideal case is 0.90±0.01 (Tr(χ)=0.69±0.01). (g) Metamaterial sample 7 (left) and an ideal partial polarizer with $T_V = 0.69 \pm 0.02$ (right). The process fidelity of the sample to the ideal case is 0.87±0.02 (Tr(χ)=0.85±0.02).

Figure S2. Distillation of highly entangled states from non-maximally entangled pure states using different metamaterial nanoantenna arrays. (a) Density matrix of the initial state of the form $|\Phi_\varepsilon\rangle = (\varepsilon|\mathrm{H}\rangle|\mathrm{H}\rangle + |\mathrm{V}\rangle|\mathrm{V}\rangle)/\sqrt{1+\varepsilon^2}$. (b)-(h), Density matrices of the distilled states from each of the seven metamaterials in ascending order. Panel h corresponds to the density matrix shown in Figure 3 in the main text.

Figure S3. Distillation of highly entangled states from non-maximally entangled mixed states using metamaterial nanoantenna arrays. (a), (e), (i): Density matrices of the three starting mixed states of the form $\rho_{\varepsilon,\lambda} = \frac{1}{1+\varepsilon^2}\left[\varepsilon^2|HH\rangle\langle HH| + |VV\rangle\langle VV| + \varepsilon\left(1-\frac{\lambda}{2}\right)(|HH\rangle\langle VV| + |VV\rangle\langle HH|)\right]$. (b), (f), (j): Density matrices of the

distilled states for the starting mixed states of a,e,i . (c), (g), (k): Density matrices of the three starting mixed states of the form $\sigma_{\varepsilon,\lambda} = \frac{1}{1+\varepsilon^2}\left[\varepsilon^2|HV\rangle\langle HV| + |VH\rangle\langle VH| + \varepsilon\left(1-\frac{\lambda}{2}\right)(|HV\rangle\langle VH| + |VH\rangle\langle HV|)\right]$. (d), (h), (l): Density matrices of the distilled states for the starting mixed states of (c), (g), (k). See Table 1 in the main text for the estimated EOF, fidelity and purity of all the starting states and distilled states (left hand column for $\rho_{\varepsilon,\lambda}$ and right hand column for $\sigma_{\varepsilon,\lambda}$ in ascending order). The panels (i), (j), (k), (l) correspond to the density matrices shown in Figure 4 in the main text.

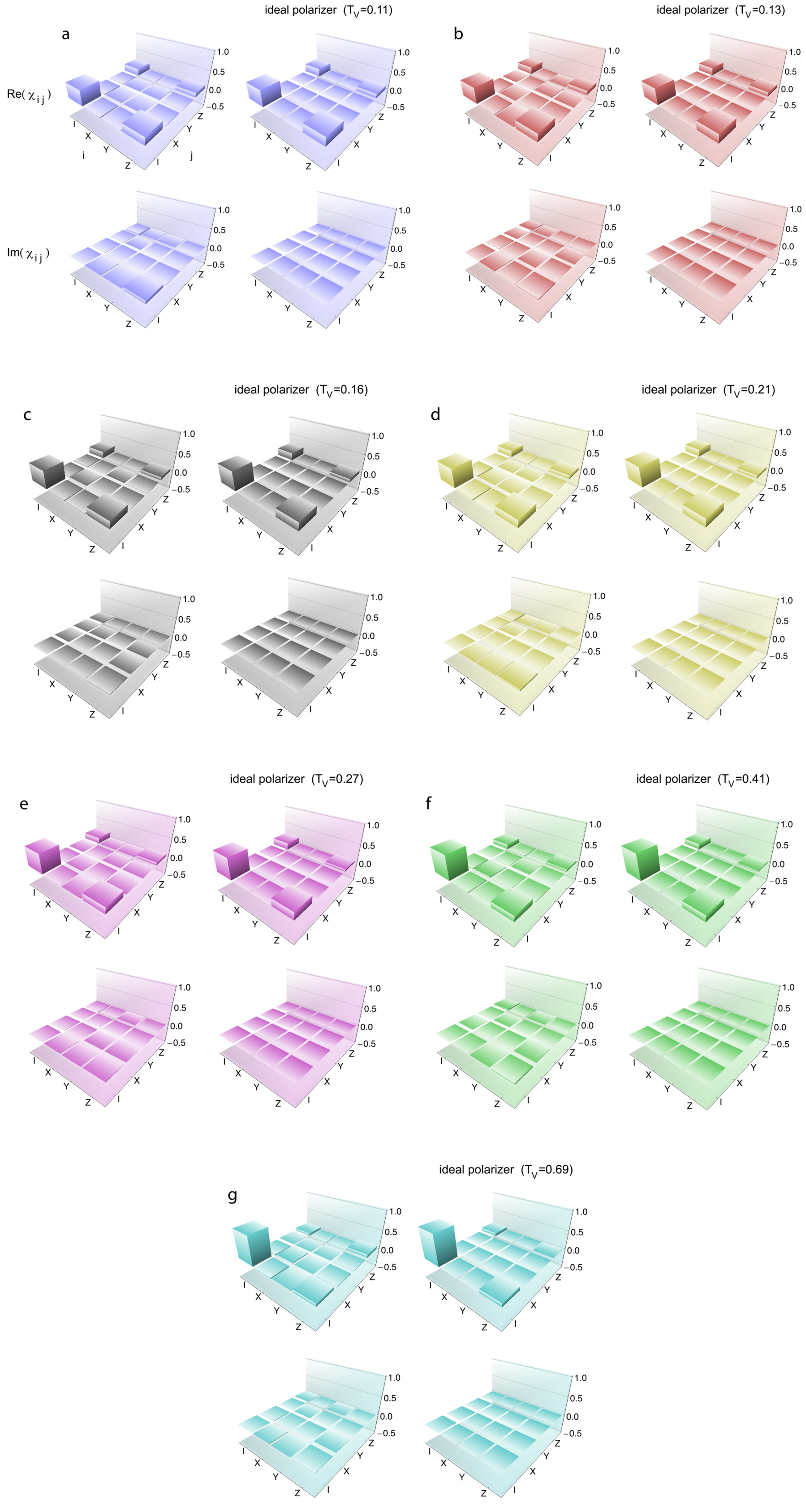
ideal polarizer (T$_V$=0.11)
ideal polarizer (T$_V$=0.13)
ideal polarizer (T$_V$=0.16)
ideal polarizer (T$_V$=0.21)
ideal polarizer (T$_V$=0.27)
ideal polarizer (T$_V$=0.41)
ideal polarizer (T$_V$=0.69)
a
b
c
d
e
f
g
Re(χ_{ij})
Im(χ_{ij})
i
j
I
X
Y
Z
1.0
0.5
0.0
−0.5

Figure S1

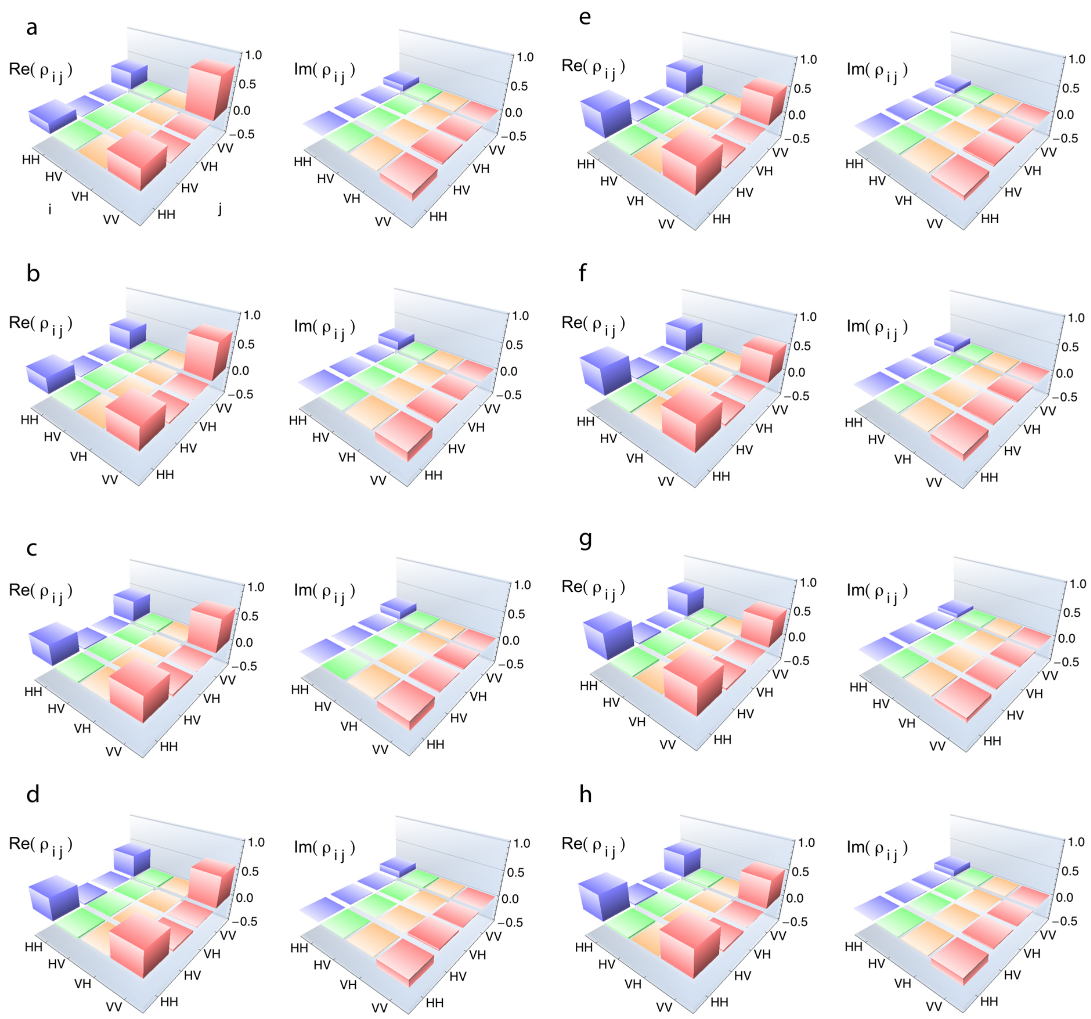

Figure S2

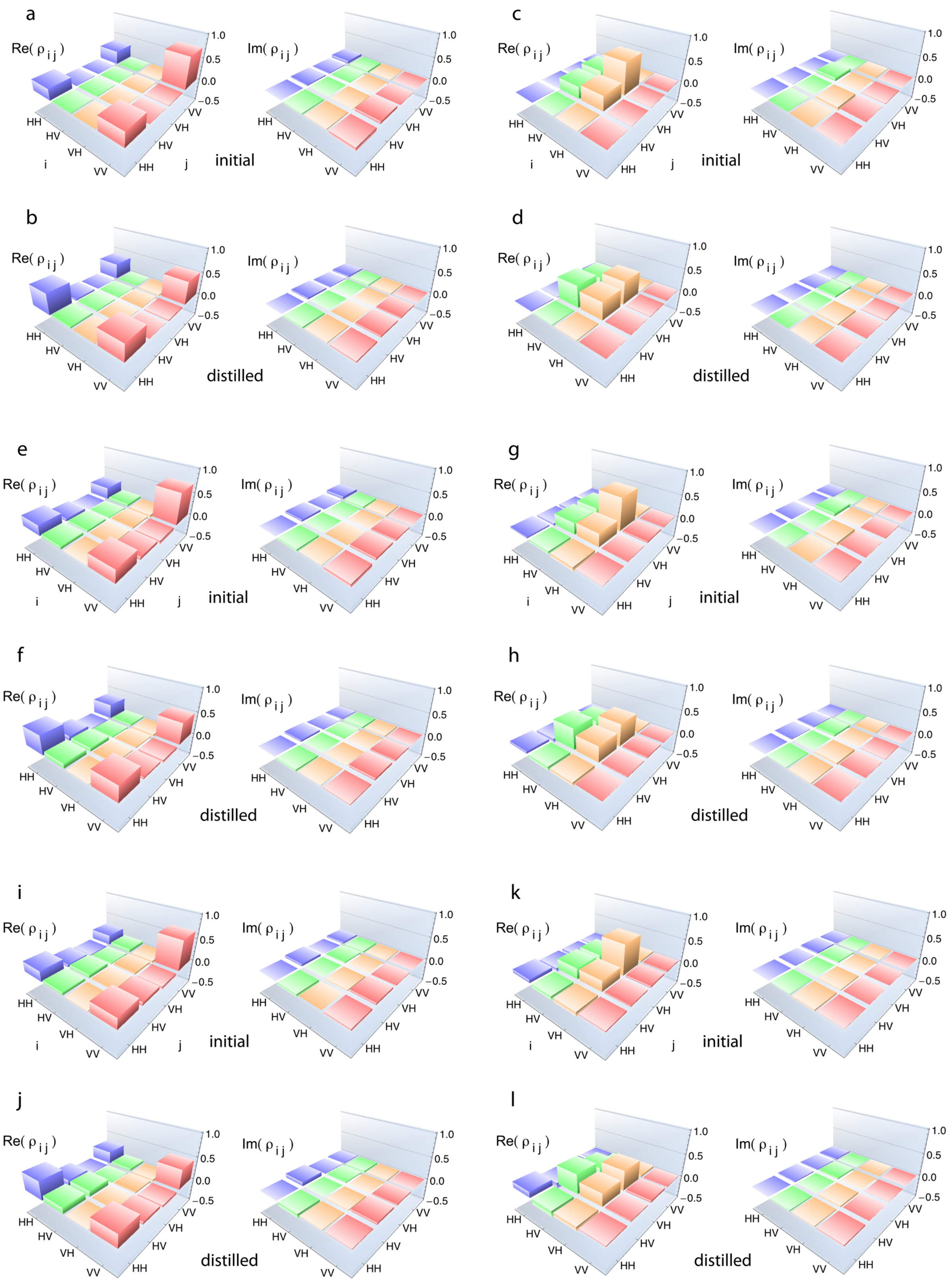
a
b
c
d
e
f
g
h
i
j
k
l
Re(ρ i j)
Im(ρ i j)
initial
distilled
HH
HV
VH
VV
i
j
1.0
0.5
0.0
−0.5

Figure S3
